# Phase Ib/II study of nivolumab combined with palliative radiation therapy for bone metastasis in patients with HER2-negative metastatic breast cancer

**DOI:** 10.1038/s41598-022-27048-3

**Published:** 2022-12-27

**Authors:** Masahiro Takada, Michio Yoshimura, Takeshi Kotake, Kosuke Kawaguchi, Ryuji Uozumi, Masako Kataoka, Hironori Kato, Hiroshi Yoshibayashi, Hirofumi Suwa, Wakako Tsuji, Hiroyasu Yamashiro, Eiji Suzuki, Masae Torii, Yosuke Yamada, Tatsuki Kataoka, Hiroshi Ishiguro, Satoshi Morita, Masakazu Toi

**Affiliations:** 1 Department of Breast Surgery, Kyoto University Graduate School of Medicine, 54 Kawaharacho, Shogoin, Sakyo-Ku, Kyoto, 606-8507 Japan; 2 Department of Radiation Oncology and Image-Applied Therapy, Kyoto University Graduate School of Medicine, Kyoto, Japan; 3 Department of Medical Oncology, Kansai Electric Power Hospital, Osaka, Japan; 4 Department of Biomedical Statistics and Bioinformatics, Kyoto University Graduate School of Medicine, Kyoto, Japan; 5 Department of Diagnostic Imaging and Nuclear Medicine, Kyoto University Graduate School of Medicine, Kyoto, Japan; 6 Department of Breast Surgery, National Hospital Organization Kyoto Medical Center, Kyoto, Japan; 7 Wakayama Breast Clinic, Wakayama, Japan; 8 Department of Breast Surgery, Hyogo Prefectural Amagasaki General Medical Center, Amagasaki, Japan; 9 Department of Breast Surgery, Shiga General Hospital, Moriyama, Japan; 10 Department of Breast Surgery, Tenri Hospital, Tenri, Japan; 11 Department of Breast Surgery, Kobe City Medical Center General Hospital, Kobe, Japan; 12 Department of Breast Surgery, Japanese Red Cross Wakayama Medical Center, Wakayama, Japan; 13 Department of Diagnostic Pathology, Kyoto University Hospital, Kyoto, Japan; 14 Department of Pathology, Iwate Medical University, Yahaba, Japan; 15 Breast Oncology Service, Saitama Medical University International Medical Center, Hidaka, Japan

**Keywords:** Medical research, Oncology

## Abstract

Radiation therapy (RT) can enhance the abscopal effect of immune checkpoint blockade. This phase I/II study investigated the efficacy and safety of nivolumab plus RT in HER2-negative metastatic breast cancer requiring palliative RT for bone metastases. Cohort A included luminal-like disease, and cohort B included both luminal-like and triple-negative disease refractory to standard systemic therapy. Patients received 8 Gy single fraction RT for bone metastasis on day 0. Nivolumab was administered on day 1 for each 14-day cycle. In cohort A, endocrine therapy was administered. The primary endpoint was the objective response rate (ORR) of the unirradiated lesions. Cohorts A and B consisted of 18 and 10 patients, respectively. The ORR was 11% (90% CI 4–29%) in cohort A and 0% in cohort B. Disease control rates were 39% (90% CI 23–58%) and 0%. Median progression-free survival was 4.1 months (95% CI 2.1–6.1 months) and 2.0 months (95% CI 1.2–3.7 months). One patient in cohort B experienced a grade 3 adverse event. Palliative RT combined with nivolumab was safe and showed modest anti-tumor activity in cohort A. Further investigations to enhance the anti-tumor effect of endocrine therapy combined with RT plus immune checkpoint blockade are warranted.

*Trial registration number and date of registration* UMIN: UMIN000026046, February 8, 2017; ClinicalTrials.gov: NCT03430479, February 13, 2018; Date of the first registration: June 22, 2017.

## Introduction

Metastatic breast cancer is generally difficult to cure. Although new therapeutic agents have improved the prognosis of metastatic breast cancer patients^1–3^, the median survival of metastatic breast cancer patients ranges from 1 to 2 years, depending on tumor subtypes and treatment responses^4–6^. HER2-directed therapies have dramatically improved the prognosis of patients with HER2-positive metastatic breast cancer^7,8^. In contrast, there is still room for improvement in the survival of patients with HER2-negative disease.

Several clinical trials have shown modest activity of immune checkpoint blockade (ICB) monotherapy in patients with metastatic triple-negative breast cancer (TNBC). In the KEYNOTE-086 trial, the objective response rate (ORR) of pembrolizumab monotherapy in patients with previously untreated, PD-L1-positive, metastatic TNBC was 21.4%, while that in previously treated, unselected, metastatic TNBC was only 5.3%^9,10^. The robust anti-tumor activity of ICB has been demonstrated in metastatic TNBC in combination with chemotherapy. Two large phase III trials demonstrated prolonged progression-free survival (PFS) among patients with PD-L1-positive metastatic TNBC who received ICB concurrently with chemotherapy in the first-line setting^11,12^. However, combination therapy with ICB and chemotherapy showed limited anti-tumor activity in patients with PD-L1-negative metastatic TNBC. ICB monotherapy showed limited anti-tumor activity in patients with estrogen receptor (ER)-positive metastatic breast cancer because of the low immunogenicity of the patient population^13^. In patients with ER-positive/HER2-negative metastatic breast cancer, ICB monotherapy showed an ORR ranging from 2.8% in the unselected population to 12% in the PD-L1-positive population^14,15^. However, significant evidences have emerged in recent years indicating immunogenic subsets of ER-positive/HER2-negative metastatic breast cancer^16^ and that ICB may be useful in combination with the appropriate therapies^17^. A systematic review showed that 43% of patients with hormonal receptor-positive breast cancer demonstrated CD8^+^ T-cell infiltration in their tumor indicating immunogenic subsets of ER-positive/HER2-negative breast cancer^16^. A randomized trial is on-going investigating pembrolizumab versus placebo in combination with neoadjuvant chemotherapy followed by adjuvant endocrine therapy in high-risk ER-positive/HER2-negative early breast cancer (MK-3475–756/KEYNOTE-756 trial, ClinicalTrials.gov Identifier: NCT03725059). A new treatment strategy to augment the anti-tumor effects of ICB in the ER-positive subtype is warranted.

Bone is one of the frequent metastatic sites of breast cancer^18,19^. Palliative radiation therapy (RT) is used for pain control of the bone metastases. RT strengthens the immune response to tumors, according to several studies^20–25^. RT provides synergistic benefits with ICBs by boosting lymphocyte infiltration into tumors, triggering immunogenic cell death, and promoting antigen-presenting cell performance^26^. Pre-clinical studies have reported that RT can induce immunogenic cell death, cytokine and chemokine production in the tumor microenvironment, the release of tumor antigens, and release of damage-associated molecular patterns, resulting in a type I interferon response and subsequent anti-tumor CD8^+^ T cell responses^27^. In addition, RT can trigger the anti-tumor effect in metastatic lesions outside of the radiation field, known as the abscopal effect, by immune-mediated mechanisms^28^. However, it has been reported that PD-L1 was upregulated in the tumor microenvironment after RT^29^, which may limit the immunomodulatory effect of RT. Several preclinical and clinical studies have indicated that RT has the potential to modulate the anti-tumor effects of ICB^30–33^. A proof-of-principal study investigated the abscopal effect of RT plus granulocyte–macrophage colony-stimulating factor to the unirradiated lesion among patients with metastatic solid tumor^30^. In this study, 36% of patients with metastatic breast cancer showed objective response to the combination therapy. A phase 3 trial assessed anti-tumor activity of ipilimumab after RT for bone metastasis (8 Gy in one fraction) in patients with metastatic castration-resistant prostate cancer that has progressed after docetaxel^31^. In this trial, the ipilimumab arm showed longer overall survival compared to the placebo arm.

This study aimed to investigate the efficacy and safety of the combination of nivolumab, anti-PD-1 antibody, and RT in patients with HER2-negative metastatic breast cancer who need palliative RT for bone metastasis. We evaluated anti-tumor effect of the combination therapy at the unirradiated target lesions.

## Results

### Study patients

**Figure 1:**
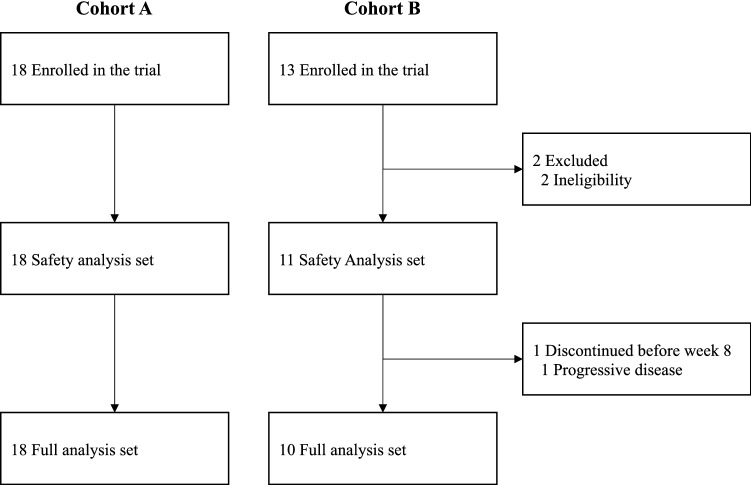
Consolidated standards of reporting trials (CONSORT) flow diagram.

The first patient was enrolled in this study on June 22, 2017. From June 2017 to November 2018, 31 patients were enrolled in this study (18 in cohort A and 13 in cohort B) (Fig. 1). Two patients in cohort B did not receive nivolumab due to ineligibility. One patient in cohort B received one dose of nivolumab but progressed before completing cycle 1. Therefore, FAS consisted of 18 patients in cohort A and ten patients in cohort B. Baseline characteristics are summarized in Table 1. The median age was 52 years (range, 35–73 years) for cohort A and 62 years (47–76 years) for cohort B. Two patients (11%) in cohort A and five patients (50%) in cohort B had a PS of 1. All patients in cohort A and eight patients (80%) in cohort B had ER/PgR-positive disease.

**Table 1:** Patient characteristics.

Factors		Cohort A (N = 18)	Cohort B (N = 10)
Age	Median (range)	52.0 (35–73)	62.0 (47–76)
ECOG PS	0	16 (89%)	5 (50%)
	1	2 (11%)	5 (50%)
ER and/or PgR	Negative	0 (0%)	2 (20%)
	Positive	18 (100%)	8 (80%)
Site of metastasis at baseline	Visceral	16 (89%)	7 (70%)
	Non-visceral	2 (11%)	3 (30%)
Liver metastasis at baseline	Yes	14 (78%)	7 (70%)
	No	4 (22%)	3 (30%)
CNS metastasis at baseline	Yes	0 (0%)	0 (0%)
	No	18 (100%)	10 (100%)
Prior endocrine therapy for metastasis	Median (range)	1 (0–2)	2.5 (0–5)
	0	2 (11%)	1 (10%)
	1	9 (50%)	2 (20%)
	2	7 (39%)	2 (20%)
	≥ 3	0 (0%)	5 (50%)
Prior chemotherapy for metastasis	Median (range)	0 (0–2)	4 (2–6)
	0	10 (56%)	0 (0%)
	1	7 (39%)	0 (0%)
	2	1 (6%)	2 (20%)
	≥ 3	0 (0.0%)	8 (80%)
Combined endocrine therapy	SERM	2 (11%)	0 (0%)
	AI + / − LH-RH analog	8 (44%)	0 (0%)
	Fulvestrant + / − LH-RH analog	8 (44%)	0 (0%)
	None	0 (0%)	0 (0%)

*ECOG PS* Eastern Cooperative Oncology Group performance status, *ER* estrogen receptor, *PgR* progesterone receptor, *CNS* central nervous system, *SERM* selective estrogen receptor modulator, *AI* aromatase inhibitor.

The median number of prior lines of endocrine therapy for metastatic disease was 1 (range, 0–2) in cohort A. In cohort A, eight patients (44%) had a history of prior chemotherapy in the metastatic setting. In cohort B, the median number of prior lines of chemotherapy for metastatic disease was 4 (range, 2–6). All patients in cohort A received endocrine therapy concurrently with nivolumab. Eight patients received aromatase inhibitor with or without LH-RH analogue, 8 received fulvestrant with or without LH-RH analogue, and two received selective estrogen receptor modulators.

None of the patients had brain metastases at the time of study entry. Sixteen patients (89%) in cohort A and seven (70%) patients in cohort B had visceral metastases. Fourteen patients (78%) in cohort A and seven (70%) patients in cohort B had liver metastases.

Tumor tissue samples from 23 out of 28 patients were available for the exploratory analysis of PD-L1 expression. The PD-L1 expression was negative in all tumor samples.

### Efficacy

**Figure 2:**
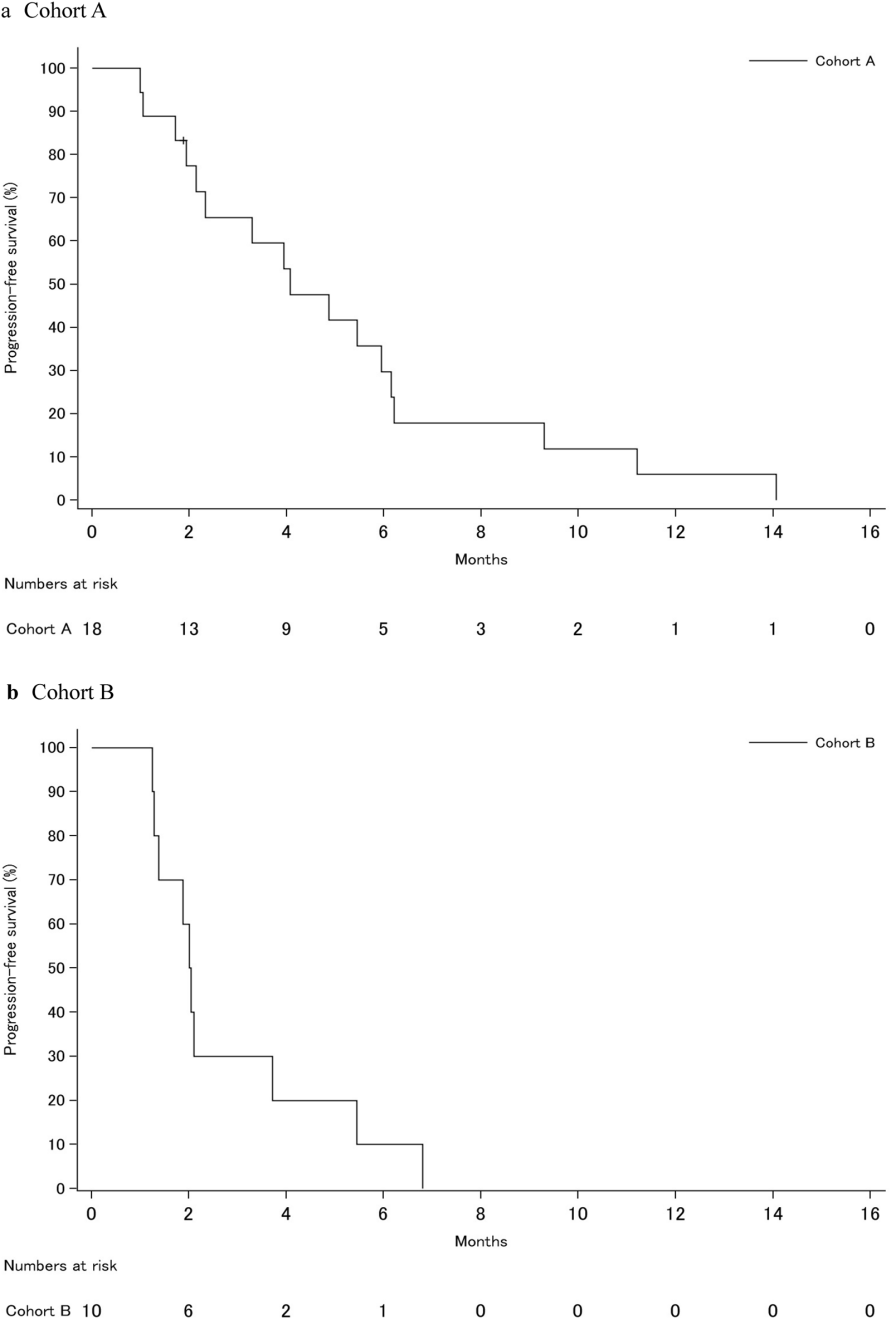
Kaplan–Meier curves of progression-free survival of the study cohort. (**a**) Cohort A, (**b**) Cohort B.

In phase Ib, no patients experienced DLT in both cohorts A and B, and both cohorts progressed to phase II. All patients received RT for one bone lesion. Two patients in cohort A and none in cohort B experienced a partial response by RECIST 1.1 in unirradiated lesions (Table 2). The ORR was 11% (90% CI 4–29%) and 0% in cohorts A and B, respectively. Five patients in cohort A experienced stable disease for ≥ 24 weeks. The DCR was 39% (90% CI 23–58%) and 0% in cohorts A and B, respectively (Fig. 2). Detailed information about irradiated bone lesions and target lesions of the seven patients who experienced clinical benefit was shown in Supplemental Table S1. Three out of seven patients received RT for bone metastasis in the pelvis. Four patients showed clinical benefit to breast or liver lesions. Tumor sample was not available in one patient who experienced stable disease. PD-L1 expression was negative in the other six patients.

**Table 2:** Anti-tumor activity assessed by RECIST v1.1.

Efficacy	Cohort A (N = 18)	Cohort B (N = 10)
ORR, N (%) [90% CI]	2 (11%) [4–29]	0 (0%) [0–21]
DCR, N (%) [90% CI]	7 (39%) [23–58]	0 (0%) [0–21]
**Best overall response, N (%)**		
Complete response	0 (0%)	0 (0%)
Partial response	2 (11%)	0 (0%)
Stable disease	5 (28%)	0 (0%)
Progressive disease	10 (55%)	7 (70%)
Could not be evaluated/assessed	1 (6%)	3 (30%)

*ORR* objective response rate, *DCR* disease control rate.

**Figure 3:**
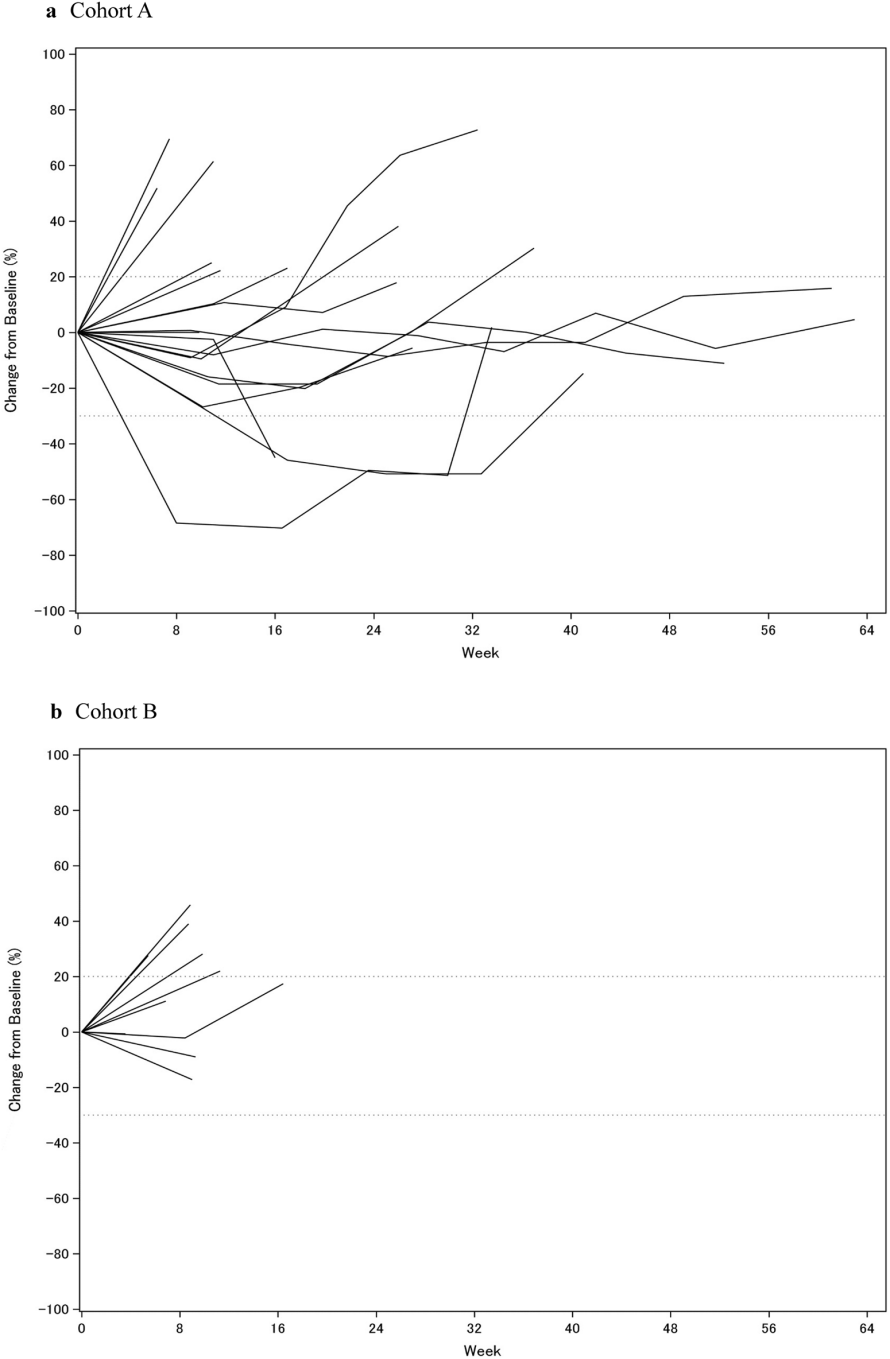
Change of sum of target lesions from baseline over time for each individual. (**a**) Cohort A, (**b**) Cohort B.

Median PFS was 4.1 months (95% CI 2.1–6.1 months) and 2.0 months (95% CI 1.2–3.7 months) in cohorts A and B, respectively (Fig. 3). The median duration of treatment with nivolumab was 23.4 weeks (range 0.1–62.1 weeks) in cohort A and 6.1 weeks (range 0.1–12.3 weeks) in cohort B.

### Safety

Ten patients (56%) in cohort A and four patients (36%) in cohort B experienced treatment-related adverse events (AEs) (Table 3). The most common treatment-related AEs of any grade included hypothyroidism (33%), hyperthyroidism (28%), rash (22%), stomatitis (11%), malaise (11%), and fever (11%). In cohort B, two patients (18%) experienced hypothyroidism. One patient in cohort B experienced treatment-related grade 3 AEs (increased aspartate aminotransferase level). None of the patients in cohort A experienced treatment-related grade 3 or more AEs. No treatment-related deaths were observed.

**Table 3:** Treatment-related adverse events.

Events	Cohort A (N = 18)		Cohort B (N = 11)	
	All grades	Grade 3–4	All grades	Grade 3–4
	N (%)	N (%)	N (%)	N (%)
Hypothyroidism	6 (33%)	0 (0%)	2 (18%)	0 (0%)
Hyperthyroidism	5 (28%)	0 (0%)	1 (9%)	0 (0%)
Rash	4 (22%)	0 (0%)	0 (0%)	0 (0%)
Stomatitis	2 (11%)	0 (0%)	0 (0%)	0 (0%)
Malaise	2 (11%)	0 (0%)	0 (0%)	0 (0%)
Fever	2 (11%)	0 (0%)	0 (0%)	0 (0%)
Diarrhea	1 (6%)	0 (0%)	1 (9%)	0 (0%)
Nausea	1 (6%)	0 (0%)	1 (9%)	0 (0%)
Abdominal pain	1 (6%)	0 (0%)	0 (0%)	0 (0%)
Colitis	1 (6%)	0 (0%)	0 (0%)	0 (0%)
Edema	1 (6%)	0 (0%)	0 (0%)	0 (0%)
GGT increased	1 (6%)	0 (0%)	0 (0%)	0 (0%)
Lymphocyte count decreased	1 (6%)	0 (0%)	0 (0%)	0 (0%)
Hyperglycemia	1 (6%)	0 (0%)	0 (0%)	0 (0%)
Headache	1 (6%)	0 (0%)	0 (0%)	0 (0%)
Pruritus	1 (6%)	0 (0%)	0 (0%)	0 (0%)
Skin induration	1 (6%)	0 (0%)	0 (0%)	0 (0%)
Fatigue	0 (0%)	0 (0%)	1 (9%)	0 (0%)
Aspartate aminotransferase increased	0 (0%)	0 (0%)	1 (9%)	1 (9%)
Alanine aminotransferase increased	0 (0%)	0 (0%)	1 (9%)	0 (0%)
Blood bilirubin increased	0 (0%)	0 (0%)	1 (9%)	0 (0%)
Alkaline phosphatase increased	0 (0%)	0 (0%)	1 (9%)	0 (0%)
Dyspnea	0 (0%)	0 (0%)	1 (9%)	0 (0%)

## Discussion

In the current study, the safety and efficacy of nivolumab combined with palliative RT for bone metastasis were evaluated in patients with HER2-negative metastatic breast cancer. In our study, endocrine therapy was co-administered with nivolumab in cohort A. Although the ORR abscopal response in this study was less than expected, clinically meaningful DCR was observed in cohort A. There were no unexpected AEs associated with combination therapy.

Three phase 2 trials have investigated the efficacy of ICB with RT in metastatic breast cancer who were unselected for PD-L1 expression. In the TONIC trial, patients with previously treated metastatic TNBC were randomly assigned to one of four cohorts with induction treatment or no induction treatment followed by nivolumab^34^. Induction cohorts consisted of RT, cyclophosphamide, cisplatin, and doxorubicin. Patients in the irradiation cohort received RT for one metastatic lesion (24 Gy in 3 fractions within ten weekdays after randomization) and then received nivolumab two weeks after randomization. The ORR evaluated by iRECIST in the irradiation cohort was 8%, which was lower than that of the cisplatin or doxorubicin cohort (23% and 35%, respectively). Ho et al. investigated the safety and efficacy of pembrolizumab with RT in patients with pretreated metastatic TNBC^35^. In their study, RT of 30 Gy was delivered in 5 fractions, and pembrolizumab was administered within three days of the first RT fraction. Approximately two-thirds of patients received RT to the soft tissue. Only three patients received RT for bone metastasis. The ORR in the unirradiated lesions was 17.6%, with three complete responses. Barroso-Sousa et al. investigated pembrolizumab with RT in patients with metastatic hormone receptor-positive breast cancer^36^. In their study, pembrolizumab was administered 2–7 days before the initiation of RT. RT consisted of 20 Gy in five fractions. Eight patients were enrolled in this study. All patients received RT for bone metastasis (one patient received RT for both bone lesions and soft tissue lesions). The median number of prior lines of chemotherapy for metastatic diseases was 2. No patients experienced objective responses, and the study was closed to further accrual.

The optimal dose and fractionation of RT combined with ICB remain unclear. In the current study, all patients received palliative RT of 8 Gy in one fraction for bone metastasis. This single-dose schedule showed equivalent efficacy to a fractionated dose (30 Gy in 10 fractions) in terms of pain relief^37^. The same single-dose RT for bone metastasis combined with ipilimumab showed a favorable safety profile and modest improvement in overall survival in patients with metastatic prostate cancer^31^. A pre-clinical study suggested that fractionated RT induced an abscopal response with a combination of anti-CTLA-4 antibody^38^. Aforementioned three phase 2 studies of metastatic breast cancer used RT doses of 20–30 Gy in 3–5 fractions. However, the ORR differed among the three studies. Further investigation is required to elucidate the optimal dose and fractionation of RT in metastatic patients treated with radioimmunotherapy.

Another important issue of ICB combined with RT is timing and sequencing. Pre-clinical studies suggested that administration of ICB before or concurrently with RT was superior to sequential administration of ICB after RT^39,40^. In the TONIC trial, nivolumab was administered two weeks after induction with hypofractionated RT in patients with metastatic TNBC^34^. Ho et al. administered pembrolizumab concurrently with RT for metastatic TNBC^35^. The difference in ORR between the two trials may be partially explained by differences in the timing and sequencing of ICB and RT. In our study, nivolumab was administered on the day after RT. This sequential schedule may explain the modest efficacy of the current study.

The RT treatment site may influence the immunogenicity of RT. McGee et al. reported that stereotactic ablative RT to the parenchymal site (lung and liver) induced a systemic immune response in peripheral blood, including an increase in activated memory CD4^+^ and CD8^+^ T cells, but RT to the non-parenchymal site (bone and brain) did not induce these changes^41^. A preclinical study also demonstrated that nodal irradiation attenuates the combinatorial efficacy of RT and ICB^42^. In the study by Barroso-Sousa et al., all eight patients received RT for bone lesions, and no objective responses were observed^36^. In the current study, all patients received RT for bone metastasis, which may explain the modest activity of combination therapy with RT and nivolumab.

Our study included only two patients with metastatic TNBC in cohort B. This number is too small to evaluate the efficacy of the combination therapy of RT and nivolumab in this patient subgroup. Most patients in our study had ER/PgR-positive, HER2-negative, metastatic breast cancer. Patients who received more than one prior chemotherapy for metastatic disease were included in cohort B. In this heavily pretreated patient cohort, there were no objective responses. Our results were compatible with the results from Barroso-Sousa et al., in which the median prior lines of chemotherapy for metastatic disease were 2. Patients in cohort A received combination therapy of RT and nivolumab in the second- or third-line setting of endocrine therapy for metastatic disease. They also received endocrine therapy concurrently with nivolumab. To the best of our knowledge, this is the first study to investigate the safety and efficacy of triplet therapy with RT, ICB, and endocrine therapy. Our study showed a modest ORR of 11% and clinically meaningful DCR of 39% in cohort A. ORR of endocrine therapy alone in second-line settings was reported to be less than 10%^43–45^. Unfortunately, it is difficult to evaluate the efficacy of combination therapy with RT and nivolumab from endocrine therapy. We plan to investigate systemic immune responses in this cohort using peripheral blood samples. Further strategies to enhance the treatment response of ICB in this patient population are warranted.

PD-L1 expression is regarded as a predictive biomarker of ICB in metastatic TNBC. In our study, 23 out of 28 patients were available for the PD-L1 analysis, and PD-L1 expression was negative in all tumor samples. Although most of patients included in our study had ER-positive disease, the results may be due to sample fixation or storage conditions. In previous studies, PD-L1 expression did not show a predictive effect for the combination therapy of RT and ICB in patients with metastatic breast cancer^34–36^. In the neoadjuvant setting, combination therapy with neoadjuvant chemotherapy and ICB showed a pathological complete response benefit regardless of PD-L1 status^46,47^. Future translational studies using tissue or blood samples will elucidate the relationship between PD-L1 expression and immune responses.

In conclusion, the combination of palliative RT for bone metastasis with nivolumab was safe and showed modest anti-tumor activity in cohort A, but not in cohort B. Further investigations regarding the optimal dose and schedule of the combination of RT and ICB, the optimal RT treatment site, the addition of other molecular targeted therapies such as PARP inhibitors or CDK4/6 inhibitors, or novel biomarkers to identify the best candidate for this treatment strategy are warranted.

## Methods

### Study design and patient population

This was a multi-institutional, multicohort, phase I/II study of nivolumab with RT in patients with HER2-negative metastatic breast cancer (UMIN: UMIN000026046; ClinicalTrials.gov: NCT03430479). This study was approved by the Institutional Review Board of Kyoto University Hospital. All participants provided written informed consent. All methods were performed in accordance with the relevant guidelines and regulations.

Eligible patients were ≥ 20 years old, had an Eastern Cooperative Oncology Group performance status (PS) of 0 to 1, and had HER2-negative metastatic disease. All patients had at least one bone metastasis candidate for palliative RT and unirradiated metastatic lesions that were measurable by Response Evaluation Criteria in Solid Tumors version 1.1^48^. Eligible patients in cohort A had ER-and/or PgR-positive invasive breast cancer, had disease progression while receiving adjuvant endocrine therapy, ≤ 12 months after endocrine therapy, or while receiving endocrine therapy for metastatic disease. Patients in cohort A must not have received more than two lines of endocrine therapy for metastatic disease and were allowed one prior line of chemotherapy for metastatic disease. Eligibility for cohort B was ≥ 2 prior chemotherapy for metastatic disease and prior history of treatment with anthracycline and taxane for primary or metastatic disease. ER-positive patients in cohort B had to be considered to have metastatic disease refractory to endocrine therapy.

Key exclusion criteria included active brain metastasis (patients with asymptomatic brain metastasis that does not require treatment could be enrolled), active autoimmune disease, history of interstitial lung disease, active diabetes, the use of systemic steroids or other immunosuppressive agents within 28 days of study entry, active infectious disease, and prior therapy with ICBs.

### Procedures

In both cohorts A and B, patients received 8 Gy of RT for bone metastasis in one fraction on day 0 (within 24 h before starting nivolumab). RT was performed to relieve the pain due to bone metastasis. The number of bone metastases to be irradiated was one or two. Nivolumab (3 mg/kg) was administered by intravenous infusion on day 1 for each 14-day cycle until progressive disease or unacceptable toxicities occurred. In cohort A, endocrine therapy of the physician’s choice was also administered from day 1.

In phase Ib, a fixed dose of nivolumab was administered, and a 3 + 3 design was used in each cohort. Dose-limiting toxicity (DLT) was defined as grade 4 neutropenia for more than seven days, grade 4 or grade 3 febrile neutropenia for more than one day, grade 4 or grade 3 thrombocytopenia requiring platelet transfusion, and grade 3 or higher non-hematological adverse events. If no patients experienced DLT, the study proceeded to phase II. If a DLT was observed in one or two out of three patients, three additional patients were included in phase Ib, and the study proceeded to phase II when less than three out of six patients experienced DLT. If all three patients experienced DLT, the cohort was terminated in phase Ib. The patients in phase Ib were also included in phase II and followed for efficacy analysis.

### Outcomes

The primary endpoint of phase Ib was the proportion of patients who experienced DLT during the first cycle of nivolumab. The primary endpoint of phase II was the ORR of unirradiated target lesions, as defined by RECIST 1.1. Secondary endpoints were duration of response (DOR), disease control rate (DCR), progression-free survival (PFS), and safety. Disease control was defined as complete response, partial response, or stable disease for ≥ 24 weeks, according to RECIST 1.1. ORR, DOR, DCR, and PFS were also evaluated using the iRECIST criteria^49^. Imaging was assessed at baseline, every eight weeks for one year, and every 12 weeks thereafter. Assessments of tumor response were performed centrally by a radiologist (MK).

An exploratory analysis of PD-L1 expression in the tumor tissue samples was conducted using anti-PD-L1 antibody (SP142 and 22C3).

### Statistical analyses

In cohort A, with 14 evaluable patients, the study had 80% power to reject the null hypothesis of ORR = 7% and an expected ORR of 30% with a one-sided significance level of 5%. Similarly, in cohort B, with 9 evaluable patients, the study had 80% power to reject the null hypothesis of ORR = 5% and an expected ORR of 30% with a one-sided significance level of 5%. Considering possible dropouts, we intended to enroll 18 and 14 patients in cohorts A and B, respectively.

Efficacy and safety analyses were based on a September 2020 database lock. Efficacy was assessed in all patients who received ≥ one nivolumab dose, had measurable disease at baseline, and underwent at least one response assessment (full analysis set: FAS). Response rates, including ORR and DCR, are reported as percentages with 90% Wilson confidence intervals (CIs). PFS was summarized as median survival time estimated using the Kaplan–Meier method with 95% CIs based on the Greenwood formula for variance derivation and log–log transformation for the survival function. Safety assessed in all patients who received at least one nivolumab dose was summarized using descriptive statistics. All analyses were performed using SAS version 9.4 (SAS Institute, Cary, NC).

## Supplementary Information

Supplementary Table S1.

## Data Availability

The data underlying this article will be shared upon reasonable request from the corresponding author. Requests for data access should be made in writing, including details of how the data will be used, and addressed to the corresponding author, and will be considered based on the scientific merit, feasibility, and timeliness of the request.

## References

[1] Chia SK (2007). The impact of new chemotherapeutic and hormone agents on survival in a population-based cohort of women with metastatic breast cancer. Cancer.

[2] Gennari A, Conte P, Rosso R, Orlandini C, Bruzzi P (2005). Survival of metastatic breast carcinoma patients over a 20-year period: A retrospective analysis based on individual patient data from six consecutive studies. Cancer.

[3] Tsuji W, Teramukai S, Ueno M, Toi M, Inamoto T (2014). Prognostic factors for survival after first recurrence in breast cancer: A retrospective analysis of 252 recurrent cases at a single institution. Breast Cancer.

[4] Greenberg PA (1996). Long-term follow-up of patients with complete remission following combination chemotherapy for metastatic breast cancer. J. Clin. Oncol..

[5] Lobbezoo DJ (2013). Prognosis of metastatic breast cancer subtypes: The hormone receptor/HER2-positive subtype is associated with the most favorable outcome. Breast Cancer Res. Treat.

[6] Fietz T (2017). Palliative systemic therapy and overall survival of 1,395 patients with advanced breast cancer - Results from the prospective German TMK cohort study. Breast.

[7] Baselga J (2012). Pertuzumab plus trastuzumab plus docetaxel for metastatic breast cancer. N. Engl. J. Med..

[8] Swain SM (2020). Pertuzumab, trastuzumab, and docetaxel for HER2-positive metastatic breast cancer (CLEOPATRA): End-of-study results from a double-blind, randomised, placebo-controlled, phase 3 study. Lancet Oncol..

[9] Adams S (2019). Pembrolizumab monotherapy for previously untreated, PD-L1-positive, metastatic triple-negative breast cancer: cohort B of the phase II KEYNOTE-086 study. Ann. Oncol..

[10] Adams S (2019). Pembrolizumab monotherapy for previously treated metastatic triple-negative breast cancer: cohort A of the phase II KEYNOTE-086 study. Ann Oncol.

[11] Cortes J (2020). Pembrolizumab plus chemotherapy versus placebo plus chemotherapy for previously untreated locally recurrent inoperable or metastatic triple-negative breast cancer (KEYNOTE-355): A randomised, placebo-controlled, double-blind, phase 3 clinical trial. Lancet.

[12] Schmid P (2018). Atezolizumab and nab-paclitaxel in advanced triple-negative breast cancer. N. Engl. J. Med..

[13] Ali HR (2015). PD-L1 protein expression in breast cancer is rare, enriched in basal-like tumours and associated with infiltrating lymphocytes. Ann. Oncol..

[14] Dirix LY (2018). Avelumab, an anti-PD-L1 antibody, in patients with locally advanced or metastatic breast cancer: A phase 1b JAVELIN Solid Tumor study. Breast Cancer Res. Treat..

[15] Rugo HS (2018). Safety and antitumor activity of pembrolizumab in patients with estrogen receptor-positive/human epidermal growth factor receptor 2-negative advanced breast cancer. Clin. Cancer Res..

[16] Stanton SE, Adams S, Disis ML (2016). Variation in the Incidence and magnitude of tumor-infiltrating lymphocytes in breast cancer subtypes: A systematic review. JAMA Oncol..

[17] Nanda R (2020). Effect of pembrolizumab plus neoadjuvant chemotherapy on pathologic complete response in women with early-stage breast cancer: An analysis of the ongoing phase 2 adaptively randomized I-SPY2 trial. JAMA Oncol..

[18] Kennecke H (2010). Metastatic behavior of breast cancer subtypes. J. Clin. Oncol..

[19] Yamashiro H (2014). Prevalence and risk factors of bone metastasis and skeletal related events in patients with primary breast cancer in Japan. Int. J. Clin. Oncol..

[20] Sauter B (2000). Consequences of cell death: exposure to necrotic tumor cells, but not primary tissue cells or apoptotic cells, induces the maturation of immunostimulatory dendritic cells. J. Exp. Med..

[21] Ho AY, Tabrizi S, Dunn SA, McArthur HL (2022). Current advances in immune checkpoint inhibitor combinations with radiation therapy or cryotherapy for breast cancer. Breast Cancer Res. Treat.

[22] Yap TA (2021). Development of immunotherapy combination strategies in cancer. Cancer Discov..

[23] Luoma AM (2022). Tissue-resident memory and circulating T cells are early responders to pre-surgical cancer immunotherapy. Cell.

[24] Katsuki S (2022). Radiation therapy enhances systemic antitumor efficacy in PD-L1 therapy regardless of sequence of radiation in murine osteosarcoma. PLoS ONE.

[25] Santa-Maria CA, Dunn SA, Ho AY (2022). Immunotherapy combined with radiation therapy in breast cancer: A rapidly evolving landscape. Semin. Radiat. Oncol..

[26] Melero I (2015). Evolving synergistic combinations of targeted immunotherapies to combat cancer. Nat. Rev. Cancer.

[27] Weichselbaum RR, Liang H, Deng L, Fu YX (2017). Radiotherapy and immunotherapy: A beneficial liaison?. Nat. Rev. Clin. Oncol..

[28] Postow MA (2012). Immunologic correlates of the abscopal effect in a patient with melanoma. N. Engl. J. Med..

[29] Deng L (2014). Irradiation and anti-PD-L1 treatment synergistically promote antitumor immunity in mice. J. Clin. Invest..

[30] Golden EB (2015). Local radiotherapy and granulocyte-macrophage colony-stimulating factor to generate abscopal responses in patients with metastatic solid tumours: A proof-of-principle trial. Lancet Oncol..

[31] Kwon ED (2014). Ipilimumab versus placebo after radiotherapy in patients with metastatic castration-resistant prostate cancer that had progressed after docetaxel chemotherapy (CA184-043): A multicentre, randomised, double-blind, phase 3 trial. Lancet Oncol..

[32] Victor CT-S (2015). Radiation and dual checkpoint blockade activate non-redundant immune mechanisms in cancer. Nature.

[33] Shaverdian N (2017). Previous radiotherapy and the clinical activity and toxicity of pembrolizumab in the treatment of non-small-cell lung cancer: A secondary analysis of the KEYNOTE-001 phase 1 trial. Lancet Oncol.

[34] Voorwerk L (2019). Immune induction strategies in metastatic triple-negative breast cancer to enhance the sensitivity to PD-1 blockade: The TONIC trial. Nat Med.

[35] Ho AY (2020). A phase 2 clinical trialassessing theefficacy and safety of pembrolizumab and radiotherapy in patients with metastatic triple-negative breast cancer. Cancer.

[36] Barroso-Sousa R (2020). A phase II study of pembrolizumab in combination with palliative radiotherapy for hormone receptor-positive metastatic breast cancer. Clin. Breast Cancer.

[37] Hartsell WF (2005). Randomized trial of short- versus long-course radiotherapy for palliation of painful bone metastases. J. Natl. Cancer Inst..

[38] Dewan MZ (2009). Fractionated but not single-dose radiotherapy induces an immune-mediated abscopal effect when combined with anti-CTLA-4 antibody. Clin. Cancer Res..

[39] Dovedi SJ (2014). Acquired resistance to fractionated radiotherapy can be overcome by concurrent PD-L1 blockade. Cancer Res..

[40] Young KH (2016). Optimizing timing of immunotherapy improves control of tumors by hypofractionated radiation therapy. PLoS ONE.

[41] McGee HM (2018). Stereotactic ablative radiation therapy induces systemic differences in peripheral blood immunophenotype dependent on irradiated site. Int. J. Radiat. Oncol. Biol. Phys..

[42] Marciscano AE (2018). Elective nodal irradiation attenuates the combinatorial efficacy of stereotactic radiation therapy and immunotherapy. Clin. Cancer Res..

[43] Baselga J (2012). Everolimus in postmenopausal hormone-receptor-positive advanced breast cancer. N. Engl. J. Med..

[44] Chia S (2008). Double-blind, randomized placebo controlled trial of fulvestrant compared with exemestane after prior nonsteroidal aromatase inhibitor therapy in postmenopausal women with hormone receptor-positive, advanced breast cancer: results from EFECT. J. Clin. Oncol..

[45] Di Leo A (2010). Results of the CONFIRM phase III trial comparing fulvestrant 250 mg with fulvestrant 500 mg in postmenopausal women with estrogen receptor-positive advanced breast cancer. J. Clin. Oncol..

[46] Schmid P (2020). Pembrolizumab for early triple-negative breast cancer. N. Engl. J. Med..

[47] Mittendorf EA (2020). Neoadjuvant atezolizumab in combination with sequential nab-paclitaxel and anthracycline-based chemotherapy versus placebo and chemotherapy in patients with early-stage triple-negative breast cancer (IMpassion031): a randomised, double-blind, phase 3 trial. Lancet.

[48] Eisenhauer EA (2009). New response evaluation criteria in solid tumours: revised RECIST guideline (version 1.1). Eur. J. Cancer.

[49] Seymour L (2017). iRECIST: Guidelines for response criteria for use in trials testing immunotherapeutics. Lancet Oncol..

