# Supplementary material for: Phase Ib/II study of nivolumab combined with palliative radiation therapy for bone metastasis in patients with HER2-negative metastatic breast cancer
Source: Sci Rep. 2022 Dec 27;12:22397. doi: 10.1038/s41598-022-27048-3 (PMC9794767; doi:10.1038/s41598-022-27048-3)
Supplement: Supplementary file 1 — Supplementary Table S1. [file 41598_2022_27048_MOESM1_ESM.docx]

**Supplemental Table S1. Detailed information of the patients who experienced clinical benefit**

| No | Cohort | Age at randomization | ER/PgR | HER2 | Combined endocrine therapy | Previous treatment lines of endocrine therapy for MBC | Previous chemotherapy for MBC | Irradiated bone lesions | Target lesions | Overall response | PD-L1 |
| --- | --- | --- | --- | --- | --- | --- | --- | --- | --- | --- | --- |
| 1 | A | 42 | positive | 0 | LR-RH analogue+letrozole | 1 | Yes | Pelvis | Liver, LN | PR | Negative |
| 2 | A | 71 | positive | 0 | Tamoxifen | 0 | No | Pelvis | Breast | PR | Negative |
| 3 | A | 65 | positive | 0 | Fulvestrant | 1 | No | Pelvis | Liver, Pleura | SD | N/A |
| 4 | A | 73 | positive | 1+ | Fulvestrant | 1 | No | Femur | Breast | SD | Negative |
| 5 | A | 69 | positive | 0 | Fulvestrant | 1 | No | Lumber spine | Breast | SD | Negative |
| 6 | A | 55 | positive | 0 | Fulvestrant | 2 | Yes | Femur | Liver, Lung | SD | Negative |
| 7 | A | 57 | positive | 0 | Exemestane | 2 | No | Lumber spine | Breast, Liver | SD | Negative |

MBC, metastatic breast cancer; LN, lymph node; N/A, not available
